# Cognitive impairment in treatment-refractory schizophrenia and type i diabetes. A case

**DOI:** 10.1192/j.eurpsy.2021.1386

**Published:** 2021-08-13

**Authors:** L. Soldado Rodriguez, S.S. Sánchez Rus, G.M. Ruiz Martinez, A. Alvarado Dafonte

**Affiliations:** 1 Mental Health Unit, Complejo Hospitalario de Jaen, Jaen, Spain; 2 Jaén, Complejo Hospitalario Jaén, Jaén, Spain

**Keywords:** Diabetes mellitus, cognitive impairment, schizophrénia

## Abstract

**Introduction:**

Even when sharing etiologic factors, the incidence of DM-1 is low in patients with schizophrenia. Both diseases can lead to cognitive impairment, but its difficult to define its origin. 33 years old male, with DM-1 and schizophrenia referred to Therapeutic Community for psychotic symptomatology control, cannabis consumption withdrawal, improvement of self-care and hipoglycemia control reach

**Objectives:**

Nowadays toxic abstinent and adequate consciousness of disorder. Remarkable persistence of hallucinations both auditive and visual, mostly shown as delirium, pharmacologic treatment-refractary. During last months, he shows excessive absent-mindedness, recent memory failure and verbal declarative memory and psychomotor slowdown Analysis: unbalance glycosylated hemoglobin. MR: cortical-subcortical atrophy, very shocking his age. Endocrinology follow up it was decided to stablish an insulin pump, so metrics were regulated.

**Methods:**

Neurological profile of the patient (deficit and slowdown attention capability) aggravation of symptoms according to glycaemia and disturbances in image test could lead to vascular origin. Attention deficit and excessive focus are symptoms of schizophrenia, but they are shown in the beginning, they tend to stabilize during years. Verbal declarative memory disruptions can be produced in both disorders

**Results:**

Better glycemic control and changed to Lurasidone 37mg and Cariprazine 3mg objecting higher reactivity and less absent-mindednes

**Conclusions:**

Cognitive impairment in DM is frequent in adults with severe and long evolving hypoglycemic episodes Regardless of its origin, the cognitive impairment in schizophrenia leads to serious impact in functional and pragmatic areas Further investigation will allow us to quantify the magnitude of cognitive effect in metabolic control so according strategies could be developed

